# GWABLUP: genome-wide association assisted best linear unbiased prediction of genetic values

**DOI:** 10.1186/s12711-024-00881-y

**Published:** 2024-03-01

**Authors:** Theo Meuwissen, Leiv Sigbjorn Eikje, Arne B. Gjuvsland

**Affiliations:** 1 Faculty of Life Sciences, Norwegian University of Life Sciences, 1432 Ås, Norway; 2 GENO SA, Storhamargata 44, 2317 Hamar, Norway

## Abstract

**Background:**

Since the very beginning of genomic selection, researchers investigated methods that improved upon SNP-BLUP (single nucleotide polymorphism best linear unbiased prediction). SNP-BLUP gives equal weight to all SNPs, whereas it is expected that many SNPs are not near causal variants and thus do not have substantial effects. A recent approach to remedy this is to use genome-wide association study (GWAS) findings and increase the weights of GWAS-top-SNPs in genomic predictions. Here, we employ a genome-wide approach to integrate GWAS results into genomic prediction, called GWABLUP.

**Results:**

GWABLUP consists of the following steps: (1) performing a GWAS in the training data which results in likelihood ratios; (2) smoothing the likelihood ratios over the SNPs; (3) combining the smoothed likelihood ratio with the prior probability of SNPs having non-zero effects, which yields the posterior probability of the SNPs; (4) calculating a weighted genomic relationship matrix using the posterior probabilities as weights; and (5) performing genomic prediction using the weighted genomic relationship matrix. Using high-density genotypes and milk, fat, protein and somatic cell count phenotypes on dairy cows, GWABLUP was compared to GBLUP, GBLUP (topSNPs) with extra weights for GWAS top-SNPs, and BayesGC, i.e. a Bayesian variable selection model. The GWAS resulted in six, five, four, and three genome-wide significant peaks for milk, fat and protein yield and somatic cell count, respectively. GWABLUP genomic predictions were 10, 6, 7 and 1% more reliable than those of GBLUP for milk, fat and protein yield and somatic cell count, respectively. It was also more reliable than GBLUP (topSNPs) for all four traits, and more reliable than BayesGC for three of the traits. Although GWABLUP showed a tendency towards inflation bias for three of the traits, this was not statistically significant. In a multitrait analysis, GWABLUP yielded the highest accuracy for two of the traits. However, for SCC, which was relatively unrelated to the yield traits, including yield trait GWAS-results reduced the reliability compared to a single trait analysis.

**Conclusions:**

GWABLUP uses GWAS results to differentially weigh all the SNPs in a weighted GBLUP genomic prediction analysis. GWABLUP yielded up to 10% and 13% more reliable genomic predictions than GBLUP for single and multitrait analyses, respectively. Extension of GWABLUP to single-step analyses is straightforward.

## Background

Best linear unbiased prediction (BLUP) was developed for the prediction of genetic values using phenotypic and pedigree information [1]. Meuwissen et al. [2] used BLUP for the prediction of single nucleotide polymorphism (SNP) effects in genomic prediction (SNP-BLUP). SNP-BLUP is equivalent to GBLUP, where genomic prediction is based on a genomic relationship matrix (**G**) and phenotypes [3]. Since GBLUP and SNP-BLUP are relatively simple to use, generally quite reliable, and easily extended to multitrait analyses, they are currently the most commonly used genomic prediction methods. In addition, they can be extended to single step predictions, which combines information from genotyped and non-genotyped individuals [4, 5].

A shortcoming of GBLUP and SNP-BLUP is that they assume that all SNPs explain an equal proportion of the total genetic variance. Based on biology, it is expected that SNPs close to major causal variants will explain more variance than others. Bayesian variable selection methods, such as BayesA, BayesB, BayesC, and BayesR [2, 6, 7], have been proposed to identify SNPs with substantial effects, and increase the variance they are expected to explain. These variable selection methods yielded substantially higher prediction accuracies in simulation studies (e.g. [8]), but in real data the increase in accuracy was often marginal (e.g. [9]). Moreover, these variable selection methods are complex and computationally very intensive, as they are typically implemented by MCMC (Monte Carlo Markov chain) sampling methods. Computationally faster, non-MCMC variable selection methods have been proposed but these are typically slightly less accurate [3, 10, 11], and since the gain in accuracy in actual data is small anyway, they may hardly improve upon GBLUP/SNP-BLUP.

One explanation for the only moderately increased selection accuracies when using Bayesian variable selection methods is that the SNP densities used (typically 50 k) are not sufficient, i.e. the SNPs are not sufficiently close to the quantitative trait loci (QTL) to find SNPs that are in very high linkage disequilibrium (LD) with the QTL. Thus, variable selection methods are expected to require higher densities to succeed. In addition, within populations with small effective sizes (Ne), the LD blocks may be large and higher SNP densities result merely in more SNPs identifying the same LD blocks. To remedy these problems, variable selection methods have been applied in multi-population datasets using high-density (HD) and whole-genome sequence (WGS) data (e.g. [7, 12]). The latter to reduce the sizes of the LD blocks that individuals have in common. This resulted in somewhat increased prediction accuracies [7, 12], but the large datasets that are required, modelling complexity, and huge computational demands have prevented large-scale practical implementations of this approach.

This approach of combining HD and WGS genotypes with large (multi-) population datasets has been successful in genome-wide association studies (GWAS), where the number of QTL detected and their mapping precision increased markedly [13]. In order to improve genomic predictions, the SNPs identifying these QTL have been added to the GBLUP or SNP-BLUP models, which increased prediction accuracies [14–17]. Here, our aim was to employ a genome-wide approach to integrate GWAS results into genomic prediction methods, i.e. instead of only including top-GWAS-SNPs with increased weights, we will base the weights of all SNPs in the GBLUP predictions on the results from a GWAS. We call this approach GWA assisted BLUP (GWABLUP), and compare it to traditional GBLUP, a Bayesian variable selection method and only adding the genome-wide significant top-SNPs to the GBLUP model. These prediction models were tested in a Norwegian Red Cattle dataset consisting of 32,201 cows with HD genotypes and recorded dairy traits (milk, fat and protein yield, and somatic cell count) in a single trait and a multitrait analysis.

## Methods

### Dataset

Individual yield deviations (YD) for the traits milk (kgMilk), fat (kgFat) and protein (kgProt) yield and log somatic cell count (SCC) on 32,201 Norwegian Red cows were provided by the cattle breeding organization GENO SA (www.geno.no) together with their birthdate. The variance components of the traits as used in the national breeding value evaluations are in Table 1. The heritabilities of these four dairy traits ranged from 0.168 to 0.306, where SCC had the lowest heritability. Since the yield deviations were averaged over a varying number of lactations, the reliability of the average yield deviation was calculated for cow $i$ as:

$${r}_{i}^{2}= \frac{{n}_{i}}{{n}_{i}+\lambda +{n}_{i}\kappa },$$

where $\lambda = V(residual)/V(genetic)$, $\kappa =V(permanent\;environment)/V(genetic)$, and ${n}_{i}$ is the number of lactations of cow $i$. Due to the differences in reliabilities, the yield deviations were weighed in all further analyses by:

1 $${w}_{i}=\frac{{n}_{i}(\alpha +1)}{\alpha +{n}_{i}},$$

where $\alpha =V(residual)/V(permanent \, environment)$. The genetic, residual and permanent environment variances, $V(genetic)$, $V(residual)$ and $V(permanent \, environment)$, respectively, are in Table 1.

**Table 1:** Genetic, permanent environmental and residual variances and heritabilities of the dairy traits

Variance component	Milk (*10^3^)	Fat	Prot	SCC
Genetic	448	642	362	0.152
Permanent environment	283	529	347	0.199
Residual	735	1782	818	0.551
Heritability	306	0.217	0.237	0.168

Table 2 shows the distribution of the number of YD over birth years, together with their average reliabilities. The birth years ranged from 1993 to 2018, but most cows were born in the 2010–2018 period. The records of the youngest cows born in 2018 (1988 cows) were chosen as validation records. The remaining 30,213 cows were used for the training of the models, i.e. they were used for the GWAS analyses and the estimation of genomic breeding values.

**Table 2:** Distribution of training and validation (in italics) animals over the birth years and the average reliabilities of their yield deviations

Birth year	Number	Reliabilities			
		kg milk	kg fat	kg protein	SCC
1993–2009	4177	0.458	0.365	0.369	0.285
2010	1072	0.476	0.383	0.384	0.300
2011	1653	0.471	0.378	0.380	0.295
2012	1767	0.464	0.371	0.374	0.290
2013	1903	0.459	0.366	0.369	0.285
2014	2322	0.444	0.350	0.356	0.273
2015	3057	0.435	0.342	0.348	0.266
2016	4938	0.418	0.325	0.334	0.253
2017	9384	0.416	0.323	0.332	0.251
*2018*	*1988*	*0.409*	*0.316*	*0.326*	*0.246*

Imputed HD genotypes on 617,739 SNPs for all 32,201 cows were provided by Geno. These imputed genotypes were a subset of a bigger dataset of imputed genotypes used in routine breeding value evaluations, where the actual genotypes came from different platforms: a customized Affymetrix 55 k SNP chip (Affymetrix, Santa Clara), Illumina BovineSNP50 BeadChip v1 and v2 (Illumina, San Diego), Illumina BovineHD Genotyping BeadChip (Illumina, San Diego) and Affymetrix 25 k (Affymetrix, Santa Clara). Genotype imputation was performed by FImpute [18]. SNPs were filtered for minor allele frequency (MAF) > 0.01, SNP call rate ≥ 0.9, Hardy–Weinberg equilibrium exact test p-values > 10^–7^, and Mendelian inconsistencies < 10%.

Centred genotype scores were obtained for cow $i$ and SNP $j$ as:

$${X}_{ij}=\left({M}_{ij}-2{p}_{j}\right),$$

where ${M}_{ij}$ are genotypes coded as 0, 1, or 2 for the homozygote, heterozygote, or opposite homozygotes, respectively; and ${p}_{j}$ is the weighted allele frequency of the SNP $j$ obtained from the training data (weights from Eq. (1)). The use of weighted allele frequencies makes that the weighted average of the genotypes ${X}_{ij}$ over the training animals is 0.

### Models for analysis

#### Genomic relationship matrices

Weighted genomic relationship matrices for the 32,201 training cows were obtained following Van Raden’s [3] method 1:

2 $${\mathbf{G}}_{\mathbf{D}}=\frac{\mathbf{X}\mathbf{D}{\mathbf{X}}^{\prime}}{\left(\sum_{j}{2p}_{j}(1-{p}_{j}){D}_{jj}\right) },$$

where $\mathbf{X}$ is a matrix of SNP genotypes ${X}_{ij}$; and $\mathbf{D}$ is a diagonal matrix of weights ${D}_{jj}$ for the SNPs $j$=1,…, 617,739. The values used as weights (${D}_{jj}$) are described below. An unweighted genomic relationship matrix ${\mathbf{G}}_{\mathbf{u}}$ is obtained by having all ${D}_{jj}$ = 1, i.e. $\mathbf{D}$ equals the identity matrix $\mathbf{I}$.

The expectation of the diagonal elements of the numerator of ${\mathbf{G}}_{\mathbf{u}}$ is $\sum_{j}{2p}_{j}(1-{p}_{j})(1+{F}_{i})$, where $(1+{F}_{i})$ and ${F}_{i}$ are the self-relationship and inbreeding coefficients of animal $i$, respectively. After dividing by the denominator $\sum_{j}{2p}_{j}(1-{p}_{j})$, the expectation of ${G}_{{u}_{ii}}$ becomes $(1+{F}_{i})$. For ${\mathbf{G}}_{\mathbf{D}}$, the expectation of the numerator is $\sum_{j}{2p}_{j}(1-{p}_{j}){D}_{jj}(1+{F}_{i})$, which after dividing by the denominator $\sum_{j}{2p}_{j}(1-{p}_{j}){D}_{jj}$ also becomes $\left(1+{F}_{i}\right)$. Hence, the two relationship matrices have the same expectation, i.e. ${\text{E}}({\mathbf{G}}_{\mathbf{u}})={\text{E}}({\mathbf{G}}_{\mathbf{D}})$, but differ from each other in real life situations due to the differences in SNP weights, which may affect variance component estimates.

#### GWAS

An efficient mixed-model association eXpedited (EMMAX)-type GWAS [19] analysis was conducted using the YD as phenotypes and the 617,739 SNP genotypes on the 30,213 training cows. The EMMAX model for the GWAS analysis of SNP $j$ is:

3 $$\mathbf{y}={\mathbf{X}}_{\mathbf{j}}{b}_{j}+\mathbf{g}+\mathbf{e},$$

where $\mathbf{y}$ is the vector of YD of the training cows; ${\mathbf{X}}_{\mathbf{j}}$ is column $j$ of the genotype matrix; ${b}_{j}$ is the effect of SNP $j$; $\mathbf{g}$ is the random effect of polygenes $\mathbf{g}\sim N\left(0,{\mathbf{G}}_{\mathbf{u}}{\sigma }_{g}^{2}\right)$ with ${\mathbf{G}}_{\mathbf{u}}$ being the unweighted $\mathbf{G}$-matrix of the training cows and; $\mathbf{e}$ is a random residual effect $\mathbf{e}\sim N\left(0,\mathbf{R}{\sigma }_{e}^{2}\right)$ with $\mathbf{R}$ being a diagonal matrix with elements ${w}_{i}^{-1}$ (see Eq. (1)). EMMAX does not re-estimate variance components per SNP, but does include an overall mean, which for simplicity was not included here. Since the weighted mean of the genotypes ${X}_{ij}$ is 0 for all SNPs $j$, the off-diagonal pertaining to the SNP-effect and the overall mean on the left-hand-side of the mixed model equations of the GWAS analysis is 0, which implies that the estimation of the SNP-effect is not affected by the overall mean. The variance components ${\sigma }_{g}^{2}$ and ${\sigma }_{e}^{2}$ were estimated using the above model excluding the SNP effect by DMU [20]. These estimates of the variance components were subsequently used in the GWAS models (3) with SNP effects.

The log-likelihood of the null-model, i.e. model (3) without the SNP effect, is:

$${L}_{0}=C-\frac{1}{2}{\mathbf{y}}^{\mathbf{^{\prime}}}{\mathbf{V}}^{-1}\mathbf{y},$$

where $C$ is a constant, and $V\left(\mathbf{y}\right)=\mathbf{V}=\mathbf{R}{\sigma }_{e}^{2}+{\mathbf{G}}_{\mathbf{u}}{\sigma }_{g}^{2}$. Letting $\widehat{{b}_{j}}$ denote the estimate of the SNP effect, the log-likelihood for the alternative model (3) with SNP effect $j$ fitted is:

$${L}_{{a}_{j}}=C-\frac{1}{2}{\left(\mathbf{y}-{\mathbf{X}}_{\mathbf{j}}\widehat{{b}_{j}}\right)}^{\prime}{\mathbf{V}}^{-1}\left(\mathbf{y}-{\mathbf{X}}_{\mathbf{j}}\widehat{{b}_{j}}\right),$$

$$L_{{a_{j} }} = C - \frac{1}{2}\left( {{\mathbf{y}}^{\prime}{\mathbf{V}}^{{ - {\mathbf{1}}}} {\mathbf{y}} - {\mathbf{y}}^{\prime}{\mathbf{V}}^{{ - {\mathbf{1}}}} {\mathbf{X}}_{\mathbf{j}} \hat{b}_{j} - \hat{b}_{j} {\mathbf{X}}^{\prime}_{{\mathbf{j}}} {\mathbf{V}}^{{ - {\mathbf{1}}}} {\mathbf{y + }}\hat{b}_{j} {\mathbf{X}}^{\prime}_{{\mathbf{j}}} {\mathbf{V}}^{{ - {\mathbf{1}}}} {\mathbf{X}}_{\mathbf{j}} \hat{b}_{j} } \right),$$

$$L_{{a_{j} }} = C - \frac{1}{2}\left( {{\mathbf{y}}^{\prime}{\mathbf{V}}^{{ - {\mathbf{1}}}} {\mathbf{y}} - {\mathbf{y}}^{\prime}{\mathbf{V}}^{{ - {\mathbf{1}}}} {\mathbf{X}}_{\mathbf{j}} \hat{b}_{j} } \right),$$

where the cancelations that led to the latter formula are due to the normal equations for the estimation of the SNP effect: ${\mathbf{X}}_{\mathbf{j}}^{\prime}{\mathbf{V}}^{-1}{\mathbf{X}}_{\mathbf{j}}\widehat{{b}_{j}}={\mathbf{X}}_{\mathbf{j}}^{\prime}{\mathbf{V}}^{-1}\mathbf{y}$. The log-likelihood-ratio (*LR*) now becomes:

$$L{R}_{j}={L}_{{a}_{j}}-{L}_{0}=\frac{1}{2}{\mathbf{y}}^{\mathbf{^{\prime}}}{\mathbf{V}}^{-1}{\mathbf{X}}_{\mathbf{j}}\widehat{{b}_{j}}$$

It may be noted that this is half the product of the right-hand-side of the normal equations for estimating the SNP effect times the estimate of the SNP effect, i.e. the *LR* is easily obtained when estimating the SNP effect. Also, since the standard-error of the SNP effect estimate is: $s{e}_{j}={({\mathbf{X}}_{\mathbf{j}}^{\prime}{\mathbf{V}}^{-1}{\mathbf{X}}_{\mathbf{j}})}^{-\frac{1}{2}}$, we have $\widehat{{b}_{j}}/s{e}_{j}={({\mathbf{X}}_{\mathbf{j}}^{\prime}{\mathbf{V}}^{-1}{\mathbf{X}}_{\mathbf{j}})}^{-\frac{1}{2}}{\mathbf{X}}_{\mathbf{j}}^{\prime}{\mathbf{V}}^{-1}\mathbf{y}$ and $L{R}_{j}=\frac{1}{2}{(\widehat{{b}_{j}}/s{e}_{j})}^{2}$, i.e. *LR* is also easily obtained from the SNP effect estimate and its standard error. In GWAS analyses, we use this log-likelihood ratio *LR* as the criterion to detect SNPs with large trait associations.

#### GBLUP

Standard GBLUP was used to predict genetic values for all cows using only records on the 30,213 training cows. The model was:

$$\mathbf{y}={\varvec{\upmu}}+\mathbf{Z}\mathbf{g}+\mathbf{e},$$

where the polygenic effect $\mathbf{g}$ was assumed randomly distributed with $\mathbf{g}\sim N(0,{\mathbf{G}}_{\mathbf{u}}{\sigma }_{g}^{2})$ and the ${\mathbf{G}}_{\mathbf{u}}$ matrix containing the genomic relationships among all the cows; and $\mathbf{Z}$ is a design matrix linking the records $\mathbf{y}$ to $\mathbf{g}$. Also, alternative GBLUP models were applied, where some top SNPs that were genome-wide-significant (P-value < 10^–7^) obtained from GWAS were given a factor 1000 more weight in the $\mathbf{G}$-matrix calculation. These analyses were denoted GBLUP(topSNPs), with genomic relationship matrices ${\mathbf{G}}_{\mathbf{t}\mathbf{o}\mathbf{p}}$, which were obtained by setting the SNP weights of the top-SNPs to ${D}_{jj}$ = 1000, while for the other SNPs ${D}_{jj}$ = 1 remained. Which SNPs were denoted as top SNPs is described in the Results section.

#### GWABLUP

In GWABLUP, the GWAS results are used to differentiate the weights of all the SNPs. First, since GWAS signals are known to be erratic and in order to mimic the modelling averaging that occurs in Bayesian variable selection models, we smoothed the $L{R}_{j}$-values by taking the moving average of the $L{R}_{j}$-values of SNP $j$ and its surrounding SNPs. E.g. let ${\overline{LR} }_{j}(5)$ denote the moving average of the $L{R}_{j}$ of five SNPs: two SNPs to the left of $j$, two to the right of $j$, and SNP $j$ itself. Moving averages of 5, 11, 21, 41, 81, and 161 SNPs were tested.

Second, posterior probabilities may be calculated using GWAS results as:

$$P{P}_{j}=\pi {e}^{{{L}_{a}}_{j}}/\left[\pi {e}^{{{L}_{a}}_{j}}+\left(1-\pi \right){e}^{{L}_{0}}\right],$$

4 $$P{P}_{j}=\pi {e}^{{LR}_{j}}/\left[\pi {e}^{{LR}_{j}}+\left(1-\pi \right)\right],$$

where $\pi$ is the prior probability that a SNP has a substantial effect, which was assumed to equal 0.001. However, instead of $L{R}_{j}$ values, we used smoothed ${\overline{LR} }_{j}()$ values as described above, which results in smoothed posterior probabilities ${\overline{PP} }_{j}({\text{s}})$, where ${\text{s}}$ is the number of SNPs involved in the moving average. Third, these smoothed ${\overline{PP} }_{j}()$ values are used as SNP weights, i.e. they are used as diagonals of the $\mathbf{D}$ matrix in Eq. (2), to calculate a weighted genomic relationship matrix, which is subsequently used in a GBLUP analysis to obtain genomic predictions. The rationale for this weighing is that the expected variance explained by the SNP equals its posterior probability times the variance explained by a SNP affecting the trait, assuming the BayesC model [6] where all SNPs with effects have equal variance. Hence, in this model, the expected variance is proportional to the posterior probability. A Julia script for the calculation of GWABLUP weights and a software for the calculation of weighted **G** matrices is available at: github.com/theomeuwissen/gghatvr4.

#### BayesGC

For comparison, we also used a Bayesian variable selection model for genomic predictions, namely the BayesGC model, which proved competitive to alternative Bayesian variable selection methods [12]. Briefly, the BayesGC model extends the above GBLUP model with a BayesC term, i.e. a term that selects and adds SNPs with large effects to the model which in addition contains a polygenic effect:

$$\mathbf{y}={\varvec{\upmu}}+\mathbf{Z}(\mathbf{g}+\sum_{j}{I}_{j}{\mathbf{X}}_{\mathbf{j}}{b}_{j})+\mathbf{e},$$

where ${I}_{j}$ is an indicator variable that indicates whether an additional effect of SNP $j$ will be fitted or not (${I}_{j}$ = 0 or 1), with a prior probability of (${I}_{j}$ = 1) of $\pi$, where $\pi =0.001$; ${b}_{j}$ is the effect of SNP $j$ with a prior distribution of ${b}_{j}\sim N(0,{\sigma }_{g}^{2}/1000)$; and the polygenic effect is a priori assumed distributed as $\mathbf{g}\sim N(0,{\mathbf{G}}_{\mathbf{u}}{\sigma }_{g}^{2})$. The BayesGC model was implemented by MCMC sampling by executing 10,000 cycles with 2000 burnin cycles in 10 replicated MCMC chains (for more details see [12]).

#### Multitrait genomic predictions

Multitrait GBLUP (MtGBLUP) predictions are obtained by using multitrait animal model theory [21], which results in 128,808 dense animal model equations. To reduce the dimensionality of the multitrait model, we used its canonical transformation [22], which is feasible since there were no missing records. The canonical transformation results in genetically and environmentally independent canonical traits that are obtained by linear combinations of the original traits and are scaled such that their environmental variances are 1. For MtGBLUP, the independent canonical traits are analysed separately using single trait GBLUP and the relationship matrix ${\mathbf{G}}_{\mathbf{u}}$. The resulting genomic estimated breeding values (GEBV) for the canonical traits are back-transformed to obtain GEBV for the original traits. The latter GEBV are the same as those obtained from the original multitrait animal model, which analyses all traits simultaneously [21].

Extension of variable selection models to multiple traits is not so straightforward as we have to decide for any QTL, which traits it affects. We used the simplifying assumption that if a SNP has substantial effects on one of the traits, it is expected to have substantial effects on all traits, i.e. its weight is increased for all traits. This assumption seems reasonable if the multitrait analysis deals with related traits, which seems to be the case here. However, variance component analysis shows that the correlations of SCC with the yield traits are rather low (see Results section).

Multitrait GWABLUP is also performed by analysis of the canonical traits. Since the canonical traits are independent, their GWAS-signals are combined by summing the ${LR}_{j}$’s of SNP $j$ across the traits, which results in an overall ${LR}_{j}$ value. Subsequently, smoothed ${\overline{LR} }_{j}(5)$ and ${PP}_{j}$ values are obtained (Eq. (4)). These ${PP}_{j}$ are used to obtain a weighted ${\mathbf{G}}_{\mathbf{D}}$-matrix which is used to analyse each of the independent canonical traits. The resulting canonical trait GEBV are back-transformed to obtain GEBV for the original traits.

The independent canonical traits were also analysed by the GBLUP(topSNPs) approach (MtGBLUP(topSNPs)). The genome-wide significant top-SNPs detected for any of the traits obtained 1000 times more weight in the ${\mathbf{G}}_{\mathbf{D}}$-matrix than the other SNPs. This ${\mathbf{G}}_{\mathbf{D}}$-matrix was used to estimate GEBV for the independent canonical traits. Similarly, single trait BayesGC analyses were applied to each of the canonical traits. The resulting GEBV for the canonical traits were back-transformed to GEBV for the original traits.

#### Comparison of methods

All the above methods were applied to the phenotypes and genotypes of the 30,213 training cows. The genotypes but not the phenotypes of the 1988 validation cows were included in the data, so that the methods also predicted their genetic values (${\widehat{\mathbf{g}}}_{\mathbf{v}}$). As a measure of the reliability of the genomic predictions we used $cor{\left({\mathbf{y}}_{\mathbf{v}},{\widehat{\mathbf{g}}}_{\mathbf{v}}\right)}^{2}$, where ${\mathbf{y}}_{\mathbf{v}}$ are the yield deviations of the validation cows. This measure of reliability is expressed relative to the reliability of the GBLUP model, which acts as a reference in the model comparisons.

The reliabilities of the alternative prediction methods are investigated for statistically significant differences (P < 0.05) by bootstrapping [23], which tests for significant differences between $cor\left({\mathbf{y}}_{\mathbf{v}},{\widehat{\mathbf{g}}}_{\mathbf{v}}^{\mathbf{k}}\right)$ and $cor\left({\mathbf{y}}_{\mathbf{v}},{\widehat{\mathbf{g}}}_{\mathbf{v}}^{{\text{l}}}\right)$, where the superscripts denote prediction method ${\text{k}}$ and ${\text{l}}$. Bootstrap samples are obtained by sampling with replacement validation individuals, i.e. their ${\mathbf{y}}_{\mathbf{v}}$, ${\widehat{\mathbf{g}}}_{\mathbf{v}}^{\mathbf{k}}$ and ${\widehat{\mathbf{g}}}_{\mathbf{v}}^{\mathbf{l}}$ values. This bootstrapping procedure accounts for the fact that both correlations are calculated using the same phenotypes $({\mathbf{y}}_{\mathbf{v}})$ and thus are not independently estimated.

Unbiasedness of BLUP breeding value estimates implies that $Cov({\widehat{\mathbf{g}}}_{\mathbf{v}},{\mathbf{g}}_{\mathbf{v}})=Var({\widehat{\mathbf{g}}}_{\mathbf{v}})$ [1], which implies that the regression coefficient ${\beta }_{{{\varvec{y}}}_{{\varvec{v}}}{\widehat{{\varvec{g}}}}_{{\varvec{v}}}}$ of ${\mathbf{y}}_{\mathbf{v}}$ on ${\widehat{\mathbf{g}}}_{\mathbf{v}}$ equals 1 (assuming no covariance between the residuals of the YD and ${\widehat{\mathbf{g}}}_{\mathbf{v}}$). Hence, these regression coefficients were estimated for the prediction methods where ${\beta }_{{{\varvec{y}}}_{{\varvec{v}}}{\widehat{{\varvec{g}}}}_{{\varvec{v}}}}=1$ implies unbiasedness, ${\beta }_{{{\varvec{y}}}_{{\varvec{v}}}{\widehat{{\varvec{g}}}}_{{\varvec{v}}}}<1$ implies that the breeding value estimates are inflated, and ${\beta }_{{{\varvec{y}}}_{{\varvec{v}}}{\widehat{{\varvec{g}}}}_{{\varvec{v}}}}>1$ that they are deflated.

## Results

### GWAS results

**Fig. 1:**
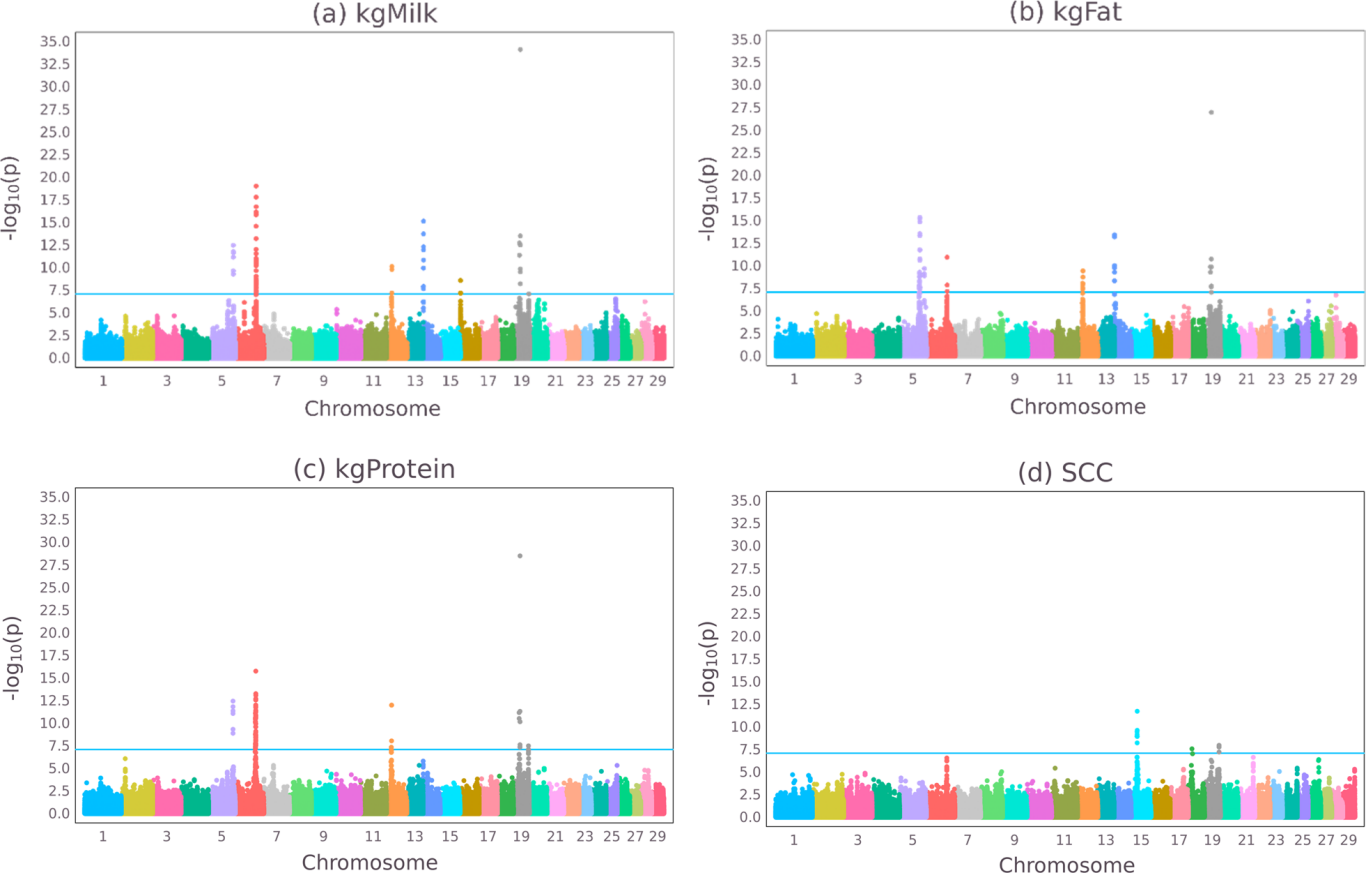
Manhattan plots of -log10(P-values) for milk (**a**), fat (**b**), and protein (**c**) yields and somatic cell count (**d**). The blue horizontal line denotes the genome-wide significance level

Figure 1 shows GWAS results in the form of Manhattan plots for the four dairy traits. Due to the large size of the dataset, some of the QTL signals were large and genome-wide significant with maximum -log10(P-values) of 34.1, 27.0, 28.5, and 11.7 for milk, fat, and protein yield and SCC, respectively. Genome-wide significant QTL were found on chromosomes 5, 6, 12, and 19 affecting the three yield traits, which agrees with the GWAS meta-analysis of [24]. The *DGAT1* gene is probably causing the signal on chromosome 14 for milk and fat yield [25], but the *DGAT1* peak is not the most significant signal due to its relatively low frequency in the Norwegian Red Cattle breed. A QTL on chromosome 12 affecting milk yield was previously detected in red cattle breeds by [26, 27].

Compared to the yield traits, SCC had less strong QTL signals with a maximum − log10(P-value) of 11.7, i.e. there seemed to be fewer major genes and thus it appeared more polygenic, which agrees with the findings of [28]. Still, there were genome-wide significant QTL on chromosomes 15, 18, and 19, which agrees with earlier findings [29, 30]. The position of the QTL on chromosome 19 seemed also to agree with the yield QTL on chromosome 19.

Based on these GWAS results of Fig. 1, in the GBLUP(topSNPs) analysis of milk, high weights (1000) were allocated to the top-SNPs on chromosomes 5, 6, 12, 14, 16, 19, which all contained genome-wide significant SNPs. For fat yield, the top-SNPs on chromosomes 5, 6, 12, 14, and 19 were included in GBLUP(topSNPs) with high weights (1000). For protein yield, GBLUP(topSNPs) gave high weights to the top-SNPs on chromosomes 5, 6, 12 and 19. And for SCC, GBLUP(topSNPs) gave high weights to the top-SNPs on chromosomes 15, 18 and 19.

**Fig. 2:**
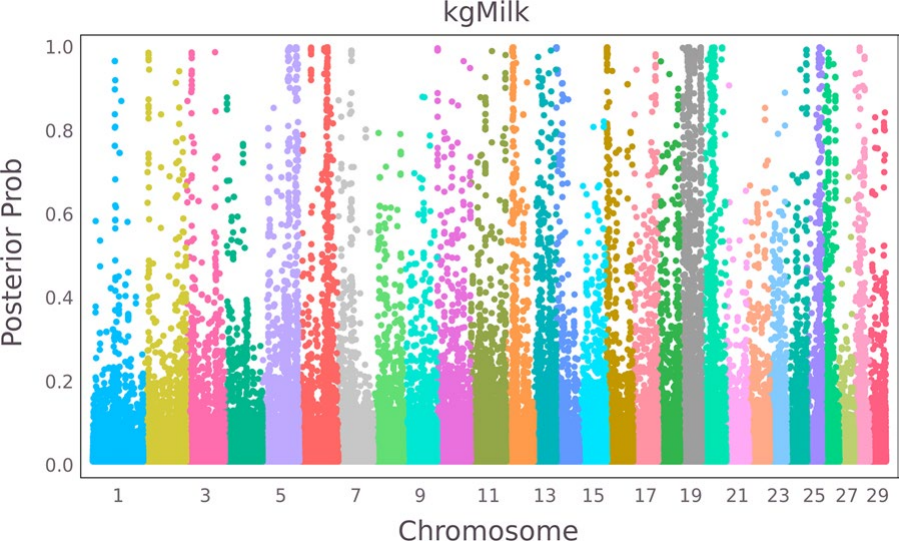
Manhattan plot of posterior probabilities of the SNPs for milk yield

For comparison, the Manhattan plot of the posterior probabilities for milk yield calculated by Eq. (4) are shown in Fig. 2. Figure 2 shows that most chromosomes contain regions with SNPs that reach posterior probabilities close to 1. The number of SNPs with posterior probabilities exceeding 0.9 was 755 out of 617,739 SNPs. Many more SNPs reached substantial posterior probabilities, and thus obtained substantial weight in the GWABLUP analysis. The number of SNPs with a posterior probability less than 0.05 was 576,113, which obtained less than 1/20th of the weight of the topSNPs in GWABLUP. However, this represents very many SNPs and their collective weight will still be substantial.

### Reliabilities of genomic predictions

Table 3 shows the ratio of the genomic prediction reliabilities of the methods for the four dairy traits relative to those of GBLUP. The $cor{\left({\mathbf{y}}_{\mathbf{v}},{\widehat{\mathbf{g}}}_{\mathbf{v}}\right)}^{2}$ of GBLUP were equal to 0.209, 0.186, 0.196, and 0.178 for milk, fat, and protein and SCC, respectively. GWABLUP reached the highest prediction reliabilities for all four traits, and yielded up to 10% more reliable predictions than GBLUP. However, GWABLUP’s reliability was only statistically significantly higher for milk and protein yield (P < 0.05). For fat yield, GWABLUP, GBLUP(topSNPs) and BayesGC were statistically significantly more reliable than GBLUP, but showed no statistically significant differences between each other. For SCC, the methods with differentially weighed SNPs, achieved only up to 1% extra reliability, confirming that SCC is a highly polygenic trait [28]. For the milk, fat and protein yield traits ${\overline{PP} }_{j}(5)$ was used, i.e. five posterior probabilities were used in the moving averages. For SCC, higher accuracies were achieved by using 81 posterior probabilities in the moving averages, which is probably due to the much weaker QTL signals for SCC (Fig. 1).

**Table 3:** Reliabilities of genomic predictions of validation cows relative to those of GBLUP and their statistically significant differences between the methods

	Reliability relative to GBLUP			
	Milk	Fat	Protein	SCC
GBLUP	1.00^a^	1.00^a^	1.00^a^	1.00^a^
GBLUP(topSNPs)	1.05^b^	1.04^b^	1.04^b^	1.00^a^
GWABLUP	1.10^c^	1.06^b^	1.07^c^	1.01^a^
BayesGC	1.05^b^	1.04^b^	1.03^b^	1.01^a^

The $cor{\left({\mathbf{y}}_{{\varvec{v}}},{\widehat{\mathbf{g}}}_{{\varvec{v}}}\right)}^{2}$ of GBLUP were 0.209, 0.186, 0.196, and 0.178 for milk, fat, and protein and SCC, respectively

Different letters in the superscripts denote statistically significant differences (P < 0.05)

The actual reliabilities of the genomic predictions can be estimated by expressing the aforementioned $cor{\left({\mathbf{y}}_{\mathbf{v}},{\widehat{\mathbf{g}}}_{\mathbf{v}}\right)}^{2}$ relative to the reliabilities of the YD of the 2018-cows (Table 2). In the case of GBLUP, this yields 0.511, 0.590, 0.603, and 0.723 for milk, fat, and protein and SCC, respectively. This shows a remarkable higher prediction reliability for SCC compared to milk yield, and fat and protein yields are in between. However, SCC has the lowest heritability (Table 1). It may be expected that the prediction reliability decreases with a decreasing heritability/reliability of the YD although the actual squared correlations $cor{\left({\mathbf{y}}_{\mathbf{v}},{\widehat{\mathbf{g}}}_{\mathbf{v}}\right)}^{2}$ are expressed relative to the reliability of the YD. The reliabilities in Table 2 may be somewhat overestimated for milk yield and somewhat underestimated for SCC. However, by expressing all the reliabilities in Table 3 relative to those of GBLUP, the results in Table 3 are not affected by any over- or underestimation of the reliabilities of the yield deviations.

### In/deflation of genomic predictions

For GBLUP, GBLUP(topSNPs), GWABLUP, and BayesGC the genomic predictions were not statistically significantly inflated (Table 4), although for GWABLUP, there was a tendency towards inflation bias for the milk, fat and protein yield traits. The latter may be because GWABLUP heavily reweighs the SNPs based on the GWAS results (Fig. 2), which are obtained from the same training data. Hence, there is a danger that the SNPs with high GWAS-based weights also show large effects in the genomic predictions, which may cause inflation bias. However, this putative inflation bias is not statistically significant in Table 4. For SCC, the moving average is calculated over much larger numbers of SNPs (81), which reduced the effects of single SNPs on the weights ${\overline{PP} }_{j}\left(81\right)$, and thus avoided any in/deflation biases.

**Table 4:** Regression coefficients (± standard errors) of the yield deviations of the validation cows on their genomic predictions

	Milk	Fat	Protein	SCC
GBLUP	1.01 $\pm$ 0.04	1.04 $\pm$ 0.05	1.02 $\pm$ 0.05	1.05 $\pm$ 0.05
GBLUP (topSNPs)	1.02 $\pm$ 0.04	1.04 $\pm$ 0.05	1.02 $\pm$ 0.05	1.04 $\pm$ 0.05
GWABLUP	0.94 $\pm$ 0.04	0.96 $\pm$ 0.04	0.93 $\pm$ 0.04	1.00 $\pm$ 0.05
BayesGC	1.00 $\pm$ 0.04	1.02 $\pm$ 0.05	1.00 $\pm$ 0.04	1.01 $\pm$ 0.05

#### Multitrait genomic predictions

The canonical transformation requires estimates of the genetic and environmental (co)variances across the traits. However, the four-trait analysis of milk, fat and protein yield and SCC did not converge using DMU. Since milk and fat yield are highly correlated, we removed fat yield from the analysis and obtained convergence for the traits milk and protein yield and SCC. The heritability estimates of milk and protein yield and SCC were: 0.26, 0.20, and 0.16, respectively (result not shown elsewhere). The genetic correlations were $$\left[\begin{array}{ccc}{r}_{g}(milk,prot)& {r}_{g}(milk,SCC)& {r}_{g}(prot,SCC)\end{array}\right]=\left[\begin{array}{ccc}0.85& 0.10& 0.096\end{array}\right]$$, and the environmental correlations were: $$\left[\begin{array}{ccc}{r}_{e}(milk,prot)& {r}_{e}(milk,SCC)& {r}_{e}(prot,SCC)\end{array}\right]=\left[\begin{array}{ccc}0.97& -0.17& 0.16\end{array}\right]$$. The canonical transformation resulted in independent canonical traits with genetic variances of 0.16, 0.29 and 1.44 for canonical traits 1, 2, and 3, respectively, and all canonical traits had standardised environmental variances of 1.

**Fig. 3:**
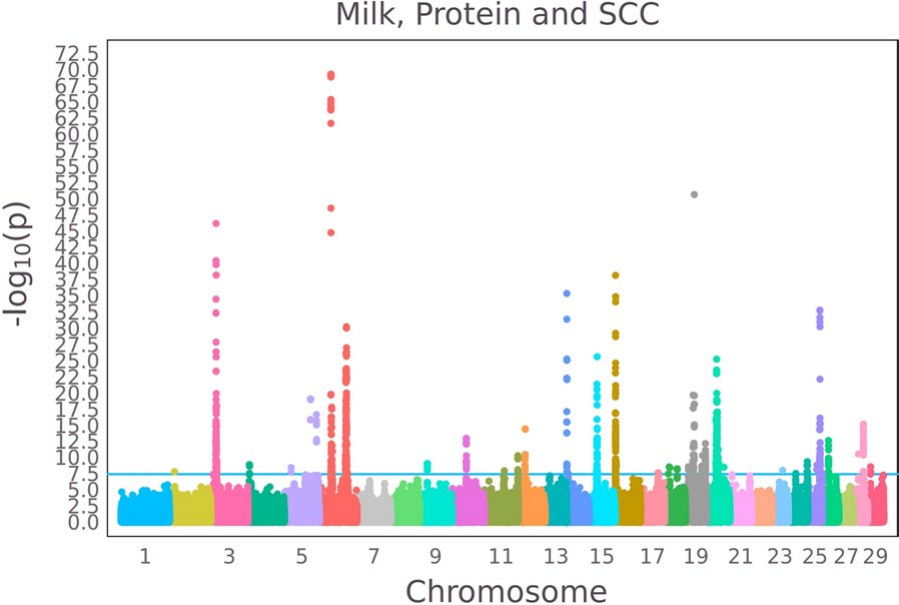
Manhattan plot of multitrait − log10(P-values) for the traits milk and protein yield and SCC

Figure 3 shows the GWAS results for milk and protein yields and SCC. Genome-wide significant SNPs were detected on chromosomes 1–6, 9–12, 14–20, 23–26, 28 and 29. Although, many of these QTL were also detected in the GWAS of the original traits, the multitrait GWAS clearly revealed more QTL, with higher levels of statistical significance. The abovementioned genome-wide significant SNPs were included in the ${\mathbf{G}}_{\mathbf{D}}$-matrix of the MtGLUP(topSNPs) analysis.

Table 5 show the reliabilities of the multitrait models relative to those of single trait GBLUP. Milk and protein yield benefitted from the multitrait analysis, but not SCC. This is probably because the aforementioned genetic correlations between the yield traits and SCC were low (≤ 0.1). The assumption that the same QTL affected all three traits (MtGBLUP(topSNPs) and MtGWABLUP) reduced the reliabilities of SCC relative to the single trait analyses, especially in the case of MtGWABLUP. The yield traits benefitted from the multitrait analyses and the MtGWABLUP – GEBV had the highest reliabilities for these traits.

**Table 5:** Reliabilities of multitrait genomic predictions of validation cows relative to those of single trait GBLUP and their statistically significant differences

	Relative reliabilities		
	Milk	Protein	SCC
MtGBLUP	1.05^a^	1.01^a^	1.00^a^
MtGBLUP(topSNPs)	1.10^b^	1.04^b^	1.00^a^
MtGWABLUP	1.13^b^	1.08^b^	0.97^a^
MtBayesGC	1.09^b^	1.04^b^	1.01^a^

The $cor{\left({\mathbf{y}}_{{\varvec{v}}},{\widehat{\mathbf{g}}}_{{\varvec{v}}}\right)}^{2}$ of GBLUP were 0.209, 0.186, 0.196, and 0.178 for milk, fat, and protein and SCC, respectively

Different letters in the superscripts denote statistically significant differences (P < 0.05)

## Discussion

A novel genomic prediction method, called GWABLUP, was proposed, and compared to three alternative genomic prediction methods in a Norwegian Red Cattle dataset comprising high-density SNP-chip genotypes and four dairy traits. In a single trait forward prediction setting, GWABLUP yielded the highest prediction accuracy for all four traits. The improved prediction accuracies of GWABLUP were statistically significant for two of the four traits. GWABLUP takes the idea of GBLUP(topSNPs), where few GWAS-based top-SNPs are fitted with high weights, to the extreme that it differentially weighs all the SNPs based on their GWAS results. To achieve the latter, it calculates posterior probabilities of the SNPs having non-zero effects using likelihood ratio statistics from the GWAS analysis, and uses smoothed posterior probabilities as weights for the calculation of a weighted genomic relationship matrix ${\mathbf{G}}_{\mathbf{w}\mathbf{a}}$. If prior biological information on the SNPs is available, e.g. some are near genes from pathways that are known to affect the trait, this can be implemented in GWABLUP by adapting the prior probabilities ($\pi$) in the ${PP}_{j}$ calculation (Eq. (4)). ${\mathbf{G}}_{\mathbf{w}\mathbf{a}}$ is subsequently used in a regular GBLUP analysis to obtain genomic predictions, that give extra weight to the most important genomic regions. Due to the equivalence of GBLUP and SNP-BLUP analyses [3], the smoothed posterior probabilities could also be used to differentiate the variances of individual SNPs in a SNP-BLUP analysis. It is also straightforward to extend GWABLUP to single step analyses (i.e. ssGWABLUP) by extending the weighted $\mathbf{G}$ matrix of Eq. (2) to an $\mathbf{H}$ matrix, that combines the relationships of genotyped and ungenotyped individuals [4].

It may seem remarkable that GWABLUP yielded higher prediction accuracies than BayesGC, which is a Bayesian variable selection method. Bayesian variable selection methods more closely resemble our biological model for complex traits, in that they assume that a large fraction of the 617,739 genome-wide SNPs are not close to causal variants and have no substantial effects, whereas a small fraction of the SNPs are near causal variants and thus show substantial effects due to their LD with these variants. Variable selection methods attempt to identify these nearby SNPs. There are some differences between Bayesian variable selection methods and GWABLUP, which may make GWABLUP more robust for external validations:

- Bayesian variable selection methods give extra weight to SNPs with effects that best explain the QTL. If a SNP is in LD with the QTL, but the QTL is better explained by another SNP, it obtains no extra weight. Thus, variable selection methods yield more precise QTL signals than single-SNP GWAS models where any SNP that is in LD with the QTL will show a likelihood ratio signal [31]. If this results in the variable selection method pointing to the correct SNP, this will be more accurate than GWABLUP. However, if the variable selection method erroneously gives all or most of the weight to a SNP that in the training data seems to explain the QTL, but in the validation data the QTL is better explained by another SNP, prediction accuracies will decrease. Because GWABLUP uses GWAS results, it will give extra weight to all SNPs that are in LD with the QTL, which may be more robust to LD changes between training and validation data.
- Bayesian variable selection and GWABLUP both weigh SNPs according to their posterior probabilities (${PP}_{j}$). The variable selection methods weigh SNP effects proportionally to $PP$, e.g. the BayesC SNP effect estimate is ${PP}_{j}$ times its BLUP estimate assuming a high SNP variance of e.g. 0.001 ${\sigma }_{g}^{2}$. In GWABLUP, the prior variances of the SNPs are reweighted proportionally to ${PP}_{j}$, which makes the information on a SNP still potentially overriding its prior variance, if it is sufficiently informative.
- Bayesian variable selection performs model averaging by averaging over alternative SNPs that are in LD with the QTL in an optimal manner. GWABLUP approximates this model averaging by using smoothed ${\overline{PP} }_{j}()$ values which are obtained as moving averages. The moving average implicitly makes use of the position of the SNPs, whereas variable selection methods do not use this information, i.e. here, GWABLUP uses more information.

In both the GWABLUP and BayesGC methods, the prior probability, $\pi$, may be varied to fine-tune genomic predictions, but this was not attempted here. Thus, although variable selection methods align more closely to our biological models for complex traits, there are a number of reasons why GWABLUP may result in more robust genomic predictions.

The GWAS analyses showed a clear difference in peak-heights across the traits, with milk yield having the highest peaks, fat and protein yield intermediate peaks, and SCC the lowest evidence for QTL (Fig. 1), although SSC still showed three genome-wide significant QTL. Top-peaks with less evidence for a QTL are probably also less accurately mapped QTL, and may indicate lower QTL signals for secondary QTL. Hence, it may be expected that GWABLUP and GBLUP(topSNPs) yield less extra genetic gain compared to GBLUP for SCC. Although, BayesGC uses a different method to position the QTL, it likely suffers as much as GWABLUP and GBLUP(topSNPs) from reduced information to clearly identify and position QTL. Smaller QTL signals may be due to the dataset being too small or to the absence of large QTL, i.e. a highly polygenic trait. In any case, a successful application of GWABLUP, GBLUP(topSNPs) and BayesGC requires the existence and accurate localization of major QTL. If the latter is not the case, GWABLUP will use a large number of SNPs in the moving average, and starts to resemble GBLUP, as is seen for SCC. Fortunately, genomic prediction datasets are often large (such as the current data), which facilitates powerful GWAS analyses.

Hence, a pre-requisite of GWABLUP is a successful GWAS, which detects and accurately maps as many QTL as possible. The success of a GWAS depends on how polygenic the trait is, and how much data are available to accurately map the QTL. A GWAS meta-analysis may be conducted [24], to combine several (across-breed) datasets to locate the QTL more accurately. A drawback of an across-breed GWAS meta-analysis is that some of the accurately detected QTL may explain less/no variance in our breed of interest, and/or the top across-breed SNP may be in lesser LD with the QTL in our breed of interest. More research is needed on the benefits of meta-GWAS studies for improving GWABLUP.

The genomic prediction methods were compared using 617 k HD SNP-chip genotypes. In the future, the use of WGS data may be envisaged. Due to the increased marker density in WGS data, it is expected that GWAS results in higher and more accurate QTL peaks since the LD between the markers and the QTL will be higher and the WGS data may even include causal polymorphisms [32]. In GWABLUP, the increased SNP-density may allow for the inclusion of more SNPs in the moving averaging process, which implies improved estimates of the posterior probabilities of the SNPs in small regions. On the contrary, the use of lower density (e.g., 50 k SNP-chips) may result in smaller QTL peaks, less opportunity for the smoothing of the QTL signals by moving averages, and thus may result in reduced prediction accuracies.

In the multitrait analysis, we assumed that all the SNPs with substantial effects are expected to affect all the traits. Hence, for every SNP $j$, one ${PP}_{j}$ across the traits is used, i.e. if the SNP is important for one of the traits, its weight will be increased for all traits. However, the actual estimate of the effect of upweighted SNPs may still be close to zero for some traits. This use of one ${PP}_{j}$ for SNP $j$ across the traits makes sense if the traits in the multitrait analysis are related. E.g. a QTL affecting milk yield may be expected to also have an effect on fat and protein yield. However, SCC was relatively uncorrelated to the yield traits, and the ${\mathbf{G}}_{\mathbf{w}\mathbf{a}}$ matrix, which was dominated by yield trait SNPs, did not improve predictions. In fact, MtGWABLUP based on this ${\mathbf{G}}_{\mathbf{w}\mathbf{a}}$ matrix resulted in less reliable SCC-predictions than single trait GWABLUP predictions, although this difference was not statistically significant.

In multitrait analyses where the traits differ substantially and the use of one ${PP}_{j}$ for SNP $j$ across the traits does not make sense, different ${\mathbf{G}}_{\mathbf{w}\mathbf{a}}$ matrices across (groups of) traits may be used. Here, every trait has its own ${\mathbf{G}}_{\mathbf{w}\mathbf{a}}$ matrix based on single-trait GWAS analyses. Also, the covariances of the individuals across the traits will require their own ${\mathbf{G}}_{\mathbf{w}\mathbf{a}}$ matrix which is obtained by using the weights ${D}_{jj}(t,s)=\sqrt{{\overline{PP} }_{j}(t){\overline{PP} }_{j}(s)}$ in Eq. (2), where ${\overline{PP} }_{j}(t) \left[{\overline{PP} }_{j}(s)\right]$ denotes the smoothed ${PP}_{j}$ for trait $t$ ($s$). More research is needed on this point and on other alternative approaches for multitrait variable selection genomic prediction.

In our data, all cows were genotyped, but in practical data this is not usually the case. Single-step methods (ssGBLUP and ssSNP-BLUP) optimally combine the information from genotyped and non-genotyped animals [4]. It is straightforward to apply GWABLUP in combination with single-step by weighing the SNPs by the smoothed posterior probabilities of the SNPs (${\overline{PP} }_{j}(t)$). The ${\overline{PP} }_{j}(t)$, which depends on ${LR}_{j}$ values (Eq. (4)), may also be obtained by combining data on genotyped and non-genotyped animals by noting that $L{R}_{j}=\frac{1}{2}{(\widehat{{b}_{j}}/s{e}_{j})}^{2}=\frac{1}{2}{t}_{j}^{2}$, where ${t}_{j}$ is the $t$ statistic of SNP $j$. Gualdron Duarte et al. [33] show how this $t$-statistic may be calculated in SNP-BLUP and ssSNP-BLUP analyses, and from GBLUP and ssGBLUP. However, obtaining prediction error variances of SNP effects will be computationally demanding in large scale single-step analyses.

Although the expectations of the unweighted and weighted genomic relationships matrices were the same, the variance component estimates using these relationship matrices might differ. For milk, fat and protein yield and SCC, the ${\mathbf{G}}_{\mathbf{w}\mathbf{a}}$ matrix yielded 9, 14, 8 and − 1% higher genetic variance estimates, respectively, than the ${\mathbf{G}}_{\mathbf{u}}$ matrix. While the 95%-confidence intervals of these estimates overlapped for all four traits, the ${\mathbf{G}}_{\mathbf{w}\mathbf{a}}$ matrix tended to result in higher genetic variance estimates, which may be due to the increased weights on the SNPs with the largest (GWAS based) effects. We did not investigate here whether using these re-estimated variance components in the GWABLUP analyses could further increased reliabilities of GEBV.

Ridge regression, which is equivalent to SNP-BLUP when applied to the estimation of SNP effects, is a well-known machine learning technique [34]. Novel genomic methods have been shown to outperform ridge regression or other classical machine learning methods when applied in fields outside genetics, such as chemometrics [35]. Since GBLUP is equivalent to SNP-BLUP [3], and our results show that GWABLUP yields more reliable prediction than GBLUP (Table 3), it may be expected that GWABLUP and its equivalent GWA-SNP-BLUP yield also more accurate predictions than ridge regression in some machine learning applications. The application of GWABLUP to general prediction problems would require first a GWAS-type of analysis to identify the likelihood ratios of x-variates (independent variates) affecting a y-variate (the dependent variate to be predicted). Second, these likelihood ratios are smoothed by calculating their moving average. If the x-variates do not come in a natural order where some variates are closer to each other than other variates, this step may be omitted. Third, these likelihood ratios are combined with the prior probability that an x-variate affects y, and are transformed into posterior probabilities using Eq. (4). Finally, a weighted ridge regression analysis will be performed where the diagonals added to the coefficient matrix are proportional to the inverse of the posterior probabilities instead of being constant.

## Conclusions

GWABLUP is based on the GBLUP or SNP-BLUP approach but weighs the SNPs according to the moving average of posterior probabilities that are based on GWAS results. In single-trait analyses, GWABLUP yielded up to 10% more reliable genomic predictions than GBLUP and yielded the highest reliability for all four traits considered here when compared to alternative methods. In a multitrait analysis, MtGWABLUP yielded up to 13% more reliable predictions, but because the ${\mathbf{G}}_{\mathbf{D}}$ matrix was dominated by yield trait QTL, MtGWABLUP predictions of SCC were less reliable than single-trait GWABLUP SCC predictions. Since its additional computations only involve a GWAS, GWABLUP is computationally considerably less demanding than Gibbs-sampling-based Bayesian variable selection methods. The latter and its straightforward extension towards single-step analyses makes GWABLUP suited to practical applications.

## Data Availability

Software are available at: github.com/theomeuwissen/gghatvr4. Data are available upon request and approval of Geno SA.
